# Scopolamine Impairs Spatial Information Recorded With “Miniscope” Calcium Imaging in Hippocampal Place Cells

**DOI:** 10.3389/fnins.2021.640350

**Published:** 2021-03-19

**Authors:** Dechuan Sun, Ranjith Rajasekharan Unnithan, Chris French

**Affiliations:** ^1^Department of Medicine, The University of Melbourne, Melbourne, VIC, Australia; ^2^Department of Electrical and Electronic Engineering, The University of Melbourne, Melbourne, VIC, Australia

**Keywords:** scopolamine, place cells, miniature microscope, calcium imaging, mAChRs

## Abstract

The hippocampus and associated cholinergic inputs have important roles in spatial memory in rodents. Muscarinic acetylcholine receptors (mAChRs) are involved in the communication of cholinergic signals and regulate spatial memory. They have been found to impact the memory encoding process, but the effect on memory retrieval is controversial. Previous studies report that scopolamine (a non-selective antagonist of mAChR) induces cognitive deficits on animals, resulting in impaired memory encoding, but the effect on memory retrieval is less certain. We tested the effects of blocking mAChRs on hippocampal network activity and neural ensembles that had previously encoded spatial information. The activity of hundreds of neurons in mouse hippocampal CA1 was recorded using calcium imaging with a miniaturised fluorescent microscope and properties of place cells and neuronal ensemble behaviour in a linear track environment were observed. We found that the decoding accuracy and the stability of spatial representation revealed by hippocampal neural ensemble were significantly reduced after the administration of scopolamine. Several other parameters, including neural firing rate, total number of active neurons, place cell number and spatial information content were affected. Similar results were also observed in a simulated hippocampal network model. This study enhances the understanding of the function of mAChRs on spatial memory impairment.

## Introduction

The formation and retrieval of episodic memory critically rely on the function of the hippocampus (Dolan and Fletcher, 1997; Fortin et al., 2002; Wiltgen et al., 2010; Carr et al., 2011). The spatial information processing ability of the hippocampus has been extensively studied, especially after the discovery of place cells by O’Keefe and Dostrovsky (1971). Place cells are hippocampal pyramidal neurons that activate in response to position and are believed to provide a spatial map of the environment (O’Keefe and Nadel’s 1978; Dragoi and Buzsaki, 2006; Moser et al., 2008; Pfeiffer and Foster, 2013). Spatial memory in rodent hippocampus is affected by acetylcholine (ACh), with muscarinic acetylcholine receptors (mAChRs) playing a significant role (Brazhnik et al., 2003; Mitsushima et al., 2013; Zhu et al., 2017). Scopolamine is a non-specific antagonist of mAChRs (Hulme et al., 1978; Lee and El-Fakahany, 1985), which has been commonly used in cognitive studies (Hayward, 2004; Mangialasche et al., 2010). Blocking mAChRs with scopolamine has been found to greatly impair spatial memory encoding (see Klinkenberg and Blokland, 2010 for review), but its effect on spatial memory retrieval is less clear. Some studies show no or very little influence of scopolamine on this (see Hasselmo, 2006 for review), while others have reported impairment (Dong Hyun and Ryu, 2008; Huang et al., 2011). We hypothesised that wide field calcium imaging of the hippocampal CA1 region would allow a more detailed understanding of the effects of mAChR on the storage and retrieval of spatial information. Previous studies have used intracranial electrode arrays to record local field potentials and single-unit activity, allowing detailed observations of cognition-related processes in terms of neural firing patterns. However, the quantity of cells and spatial accuracy are generally limited. We have therefore used *in vivo* calcium imaging to record the activity of neural ensembles in the hippocampal CA1 region of freely running mice with a miniaturised microscope (miniscope, Ghosh et al., 2011; Aharoni et al., 2019), allowing the simultaneous recording of calcium activity of a large population of neurons. We demonstrated that scopolamine greatly impaired the spatial accuracy of place cells with mice traversing a linear track, attributable to impaired stability of spatial representation revealed by hippocampal neural ensemble. Several parameters including neural firing rate, spatial information, total neuron number were affected. We found similar results in a conductance-based hippocampal network with scopolamine effects modelled by disinhibition of muscarinic modulation of voltage gated potassium channels.

## Materials and Methods

All surgical and experimental procedures were approved by the Florey Animal Ethics Committee (No. 18-008UM) and were conducted in strict accordance with the Australian Animal Welfare Committee guidelines.

### Subjects

Five naive adult male C57BL/6 mice aged 12 weeks were obtained from WEHI (Melbourne, VIC) and housed in the Biological Research Facility of the Department of Medicine, University of Melbourne. All animals weighed 24–25 g (24.66 g ± 0.10 g) at the time of surgery and were housed individually. The facility was maintained on a 12–12 h light-dark schedule (lights on: 7:30 am to 7:30 pm) with water and standard mice chow *ad libitum*.

### Drug Administration

Scopolamine hydrobromide (Sigma-Aldrich, United States) was dissolved in sterile 0.9% saline and injected intraperitoneally at a volume of 1 mg/kg (Moosavi et al., 2018).

### Stereotaxic Surgery

The surgical procedures comprised two components: viral infusion and grin lens implantation.

#### Virus Infusion

**FIGURE 1:**
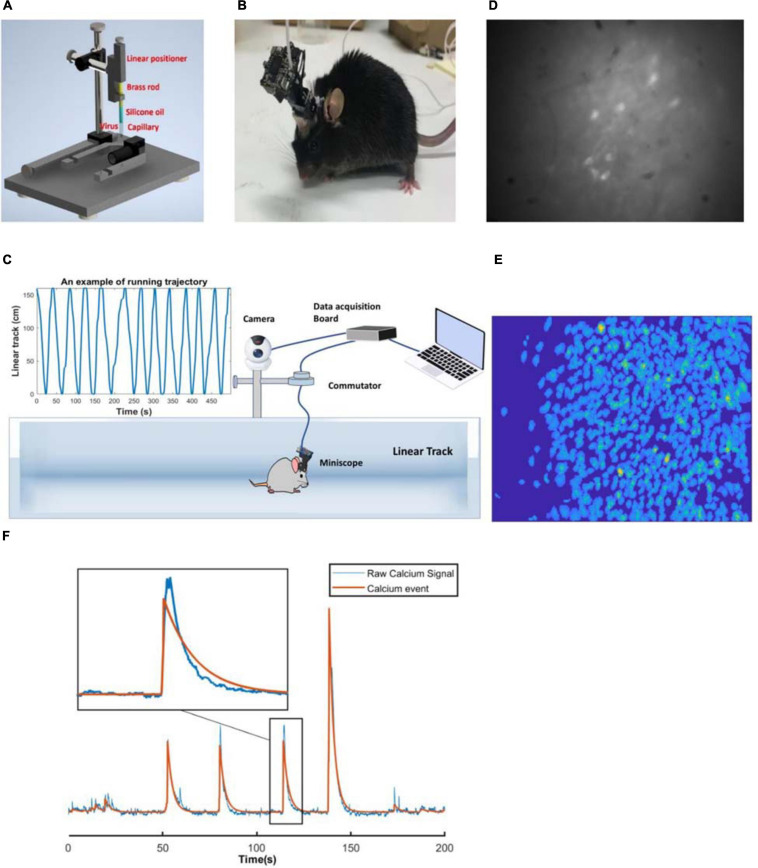
**(A)** Custom made virus infusion instrument. **(B)** An example of the mouse with the miniscope. **(C)** Linear track and miniscope recording system. **(D)** An example of the raw image. **(E)** An example of footprints of the cells identified with CNMF-E. **(F)** An example of the raw calcium signal and calcium events of a selected neuron.

The pAAV.Syn.GCaMP6f.WPRE.SV40 virus (500 nL, viral load: 2.2 × 10^13^ GC/ml; AddGene, United States) was injected into the dorsal hippocampus (AP −2.1, ML +2.1, DV −1.7 relative to bregma) with a custom made injecting system over 15 min. The virus was first loaded into a capillary (interior diameter: 0.9 mm), which was fabricated on a Sutter P-1000 electrode puller to produce a tip diameter of 20–50 μm; this was sealed with silicon oil (Sigma-Aldrich, United States) at the open end; and a round brass rod (diameter: 0.8 mm; Albion Alloys, United Kingdom), connected to 3D positioner was fitted into the capillary to control the volume of the virus injected (Figure 1A). The virus injector was left in place for an additional 10 min to allow for viral diffusion. The animal was then left for one week to recover and to allow fluorophore expression.

#### GRIN Lens Implantation

Two 1 mm screws were implanted (AP +1.8, ML −2.5; AP −2.8, ML −0.8) to serve as anchors. A small window of skull was removed by using a 2 mm drill bur, centred at AP −2.1, ML +1.6, and the exposed dura was cleaned with fine tweezers. A 27-gauge blunt needle was used to aspirate cortex to expose the vertical striations of the hippocampal fimbria, with artificial cerebrospinal fluid flowing during the procedure to provide a clear operating field. Using the most posterior point of the edge of the drilled hole (next to lambda side) as a reference, the grin lens (0.25pitch, #64-519, Edmund Optics) was implanted 1.35 mm deep, touching the surface of exposed tissue (Cai et al., 2016). Cyanoacrylate glue was applied surrounding the lens to prevent movement and dental cement was built over the glue for support. The lens was then covered with fast setting silicone adhesive (Dragon Skin^®^ Series, United States). After the surgery, the animal was injected with carprofen (5 mg/kg) and dexamethasone (0.6 mg/kg, Sigma-Aldrich, United States) intraperitoneally every day for the relief of pain and inflammation, and provided with enrofloxacin water (1:150 dilution, Baytril^®^, United States) for one week. Four weeks later, a small metal baseplate was mounted on the animal’s head to support the miniscope, which was locked in the position at the optimal focal distance. Figure 1B shows a mouse with a miniscope.

### Animal Training

After surgery, the animals were handled approximately 10 min twice a day in the daytime and weighed after each handling session for five days. A food restriction regimen was implemented to keep the animal at 85% of its original weight. The animal was then trained to run back and forth on a 1.6 m linear track for two weeks with clues painted on the walls for food reward while wearing the miniscope. On each training day, the mice performed 30 trials and a small food pellet was awarded once it could rapidly run through the track without wandering.

### Experimental Procedures

All the recordings were performed during the daytime. The animal was brought into a silent recording room 30 min before the start of recording to acclimate to the surrounding environment. After mounting the miniscope, the animal was moved to the linear track to explore the space for 30 min freely. The linear track was cleaned with 80% ethanol to eliminate scent clues. Twelve running trials were recorded as the baseline control. Imaging frames were recorded with custom-made miniscope acquisition software, with a sampling rate of 30 FPS. The animal was then injected with saline or scopolamine, replaced in its cage for 20 min and then performed another 12 running trials. A camera fixed overhead was synchronised with the miniscope to record the animal’s position. The miniscope cable was suspended over the linear track through a custom-made commutator (Figure 1C). Figure 1D shows an example of the raw fluorescent signals.

### Processing of Calcium Imaging Data

#### Image Pre-processing and Calcium Activity Deconvolution

A non-rigid motion correction algorithm was applied first to implement image registration (Pnevmatikakis and Giovannucci, 2017). Constrained Non-negative Matrix Factorisation for micro-endoscopic data (CNMF-E) was then utilised to identify and extract each neural spatial boundary and calcium activity (Zhou et al., 2018; Figure 1E). A fast deconvolution algorithm was finally applied to deconvolve the calcium activity to estimate neural spike-activity (Friedrich et al., 2017). An example of the raw calcium signal and calcium events of a selected neuron is shown in Figure 1F and Supplementary Video 1.

#### Place Field Map

The position of the animal’s head and running speed were detected using a custom Matlab script. We analysed the data of both left-to-right (LR) and right-to-left (RL) running directions separately. To analyse the neural spatial spike activity, the linear track was divided into several 2 cm spatial bins (the bins on each end were discarded). A speed range threshold was set between 8 cm/s to 25 cm/s. The neural temporal calcium event rate (the number of spikes in each bin) and the occupancy of the animal were counted and smoothed with a Gaussian smoothing kernel (σ = 1.5, size = 5). The place field map for each neuron was measured by dividing neural smoothed spatial spike activity by the smoothed bins occupancy, with the maximum value defined as the place field’s position (Rubin et al., 2015).

#### Spatial Information Content and Place Cells

The neural spatial information content (SIC) was defined as (Ravassard et al., 2013):

$$I=\sum_{i=1}^{K}P_{i}\,\frac{\lambda_{i}}{\bar{\lambda }}\,\log_{2}\frac{\lambda_{i}}{\bar{\lambda }}$$

K represents the number of bins; *P*_*i*_ is the occupancy ratio of the ith bin; λ_*i*_ is the neural calcium event rate in the ith bin; $\bar{\lambda }$ is the mean calcium event ($\sum_{i=1}^{K}P_{i}\lambda {}_{i}$).

We first calculated the spatial information content for each neuron, and then shuffled the animal’s position as well as neural temporal spike activity 1000 times. A place cell was defined as the neuron whose spatial information content was above chance (*p* < 0.05) with respect to the shuffling results.

#### Odd & Even Trials Population Vector Overlap

To quantify the stability of spatial representation revealed by hippocampal neural ensemble, we calculated the population vector overlap (PVO) between odd and even trials (Ravassard et al., 2013). The PVO is a measure of firing patterns’ similarity across locations on the linear track. A high PVO value indicates similar firing patterns at two locations.

$$P\,V\,O\,\left(x,y\right)=\frac{\sum_{n=1}^{N}\lambda_{n}\,\left(x\right)\,\lambda_{n}\,\left(y\right)}{\sqrt[{2}]{\sum_{n=1}^{N}\lambda_{n}\,\left(x\right)\,\lambda_{n}\,\left(x\right)}\cdot \sqrt[{2}]{\sum_{n=1}^{N}\lambda_{n}\,\left(y\right)\,\lambda_{n}\,\left(y\right)}}$$

*N* represents the total number of neurons; *x* and *y* represent the animal’s location bin in odd and even trials respectively; _*n*_ is the place field map of the nth neuron at different location bins.

The diagonal index (DI) is used to quantify the “diagonalisation feature” of the PVO map. A larger value indicates more evident “diagonalisation feature.”

$$D\,I=\frac{1}{\sqrt{\sum_{x}\sum_{y}{\left[P\,V\,O\,\left(x,y\right)-I\right]}^{2}}}$$

*x* and *y* represent the animal’s location bin in odd and even trials respectively;*I* is an identity matrix (diagonal elements equal to one).

#### Autoencoder

An autoencoder is a type of artificial neural network that learns how to compress and reconstruct data in an unsupervised way. It is a powerful tool for dimensionality reduction (Kramer, 1991). An autoencoder comprises an encoder and a decoder. We implemented and trained an autoencoder model by using the TensorFlow platform (Abadi et al., 2016). The encoder and decoder were both defined with three “Dense Layers” with “scaled exponential linear unit” activation function (100 nodes in the last layer of encoder). The whole model was compiled with a binary cross-entropy loss function and Adam optimiser. We used a grid search method to fit the parameters including learning rate, epochs and nodes for each animal to minimise the reconstructed data error. We wanted to explore if there were differences before and after mAChRs blockade that were reflected on the neural ensemble level. The calcium spike trains of the same neurons at baseline and saline/scopolamine were split into 2.5 s epochs and fed into the model. Principal component analysis of the encoder analysis was applied for better visualisation.

#### Decoding

A naive Bayesian decoder was utilised to estimate the position of the animal based on neuronal temporal spike activity (Davidson et al., 2009). The decoding error was defined as the average distance between the real and estimated position at each time frame.

$$P\,\left(x|n\right)=P\,\left(x\right)\,\left(\prod_{i=1}^{N}f_{i}\,{\left(x\right)}^{n_{i}}\right)\cdot \text{exp}\,\left(-\tau \,\sum_{i=1}^{N}f_{i}\,\left(x\right)\right)$$

The symbol *n* is the current input temporal spike activity; *x* is the index of the location bin; *P(x)* is the occupancy ratio of the bin x; *f*_*i*_(*x*) is the average calcium event rate of the ith neuron at bin *x*; τ is the time window length of the input temporal spike activity.

#### Hippocampal CA1 Network Model

We sought to explore the experimental results in a conductance based CA1 network derived from models previously described by Turi et al. (2019) and Shuman et al. (2020) that composed of 130 pyramidal neurons (PYR), 8 basket cells (BC), 2 axoaxonic cells (AAC), 2 bistratified cells (BIS), 2 oriens lacunosum-moleculare cells (OLM), 1 vasoactive intestinal peptide-cholecystokinin cell (VIP-CCK) and 4 vasoactive intestinal peptide–calretinin cells (VIP-CR) with the same neural characteristics and structures. Additionally, we added an M-channel conductance on the models of somatostatin positive (SST+) and parvalbumin positive (PV+) interneurons with the conductance of 2 *ps*/*um*^2^(Lawrence et al., 2006). The network connectivity is shown in Figure 10A, and the afferent inputs to CA1 stored in CA3 were simulated by applying a spatial filter on the grid-like inputs from the entorhinal cortex (Turi et al., 2019). We hypothesised that a major effect of scopolamine would be the enhancement of the muscarinic inhibited M-current that is known to strongly modulate neuronal firing rates both in pyramidal neurons and interneurons (see section “Discussion”). To simulate the effects of scopolamine, the M-channel conductances of pyramidal cells were increased by five (×5), ten (×10), fifteen (×15) and twenty (×20) times separately. The M-channel conductance amplitude of SST+ and PV+ interneurons were set at a level reproducing the neural firing reduction observed experimentally.

#### Statistics

Statistical analyses were performed using SPSS (IBM) and GraphPad Prism software. The level of significance was set at *p* < 0.05. Statistical significance was assessed by two-tailed paired Student’s *t*-test, one-way and two-way repeated-measures analysis of variance (ANOVA). Normality of the data was confirmed by Kolmogorov–Smirnov and Shapiro-Wilks test. Bonferroni correction was performed for the post hoc comparisons. No statistical methods were used to predetermine sample sizes, but our sample sizes were similar to those reported previously (Royer et al., 2012; Shuman et al., 2020). All results are shown as mean ± standard error of mean (SEM) of the percentage variation from baseline.

## Results

We compared the firing patterns of the place cell ensembles between control and treatment groups and measured total cell numbers, place cell numbers, neural firing rate, place field map, spatial information, odd and even trials PVO, decoding error, as well as the animal’s running speed. All results are shown as a percentage change following injection of saline or scopolamine relative to baseline.

### Scopolamine Reduced the Total Cell Number of Cells Detected and the Neural Firing Rate

**FIGURE 2:**
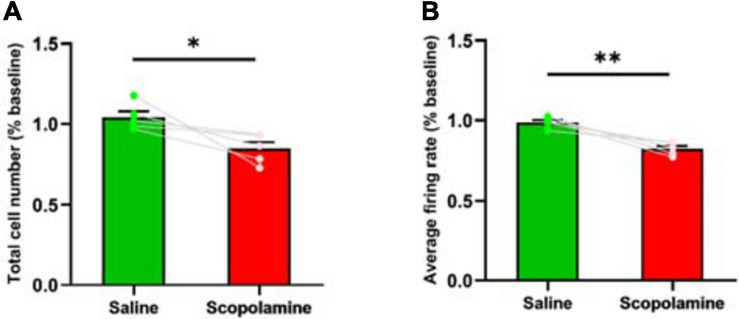
**(A)** The total neuron number within the field of view. The scopolamine treated mice had less detected neurons. **(B)** The neural average firing rate. The scopolamine treated mice had lower neural firing rate. ^∗^*P* < 0.05, ^∗∗^*P* < 0.01 represent the significant difference between scopolamine and saline groups.

We first studied the effects of blocking mAChRs on the number of detected neurons and the neural firing rate. Scopolamine significantly reduced the number of detected neurons (84.7% ± 9.10% relative to baseline) compared with saline (101.8% ± 1.45% relative to baseline; paired t-test, *P* = 0.048; Figure 2A). Prior to the injection of scopolamine, there were 640 ± 46 neurons recorded in each animal, decreasing to 541 ± 46 following injection. Additionally, we found that the scopolamine reduced the neural firing rate to 83.39% ± 2.22% relative to baseline. This was significantly lower than saline controls with 98.72% ± 1.50% relative to baseline (paired *t*-test, *P* = 0.006; Figure 2B). Prior to the injection of scopolamine, the average neural firing rate was 0.077 ± 0.012 Hz, and it decreased to 0.063 ± 0.009 Hz after the administration of scopolamine.

### Reduction of Place Cell Number and Spatial Information Content Occurred With Scopolamine Administration

**FIGURE 3:**
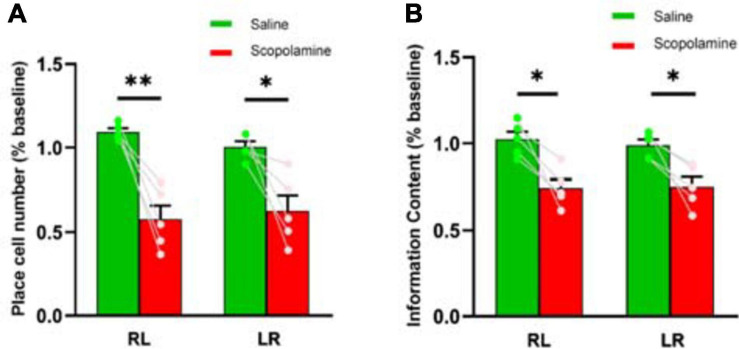
**(A)** The place cell number detected during both right-to-left (RL) and left-to-right (LR) running trials. The scopolamine treated mice had less place cells **(B)** The neural spatial information content measured during both RL and LR running trials. The scopolamine treated mice had lower information content. ^∗^*P* < 0.05, ^∗∗^*P* < 0.01 represent the significant difference between scopolamine and saline groups.

**FIGURE 4:**
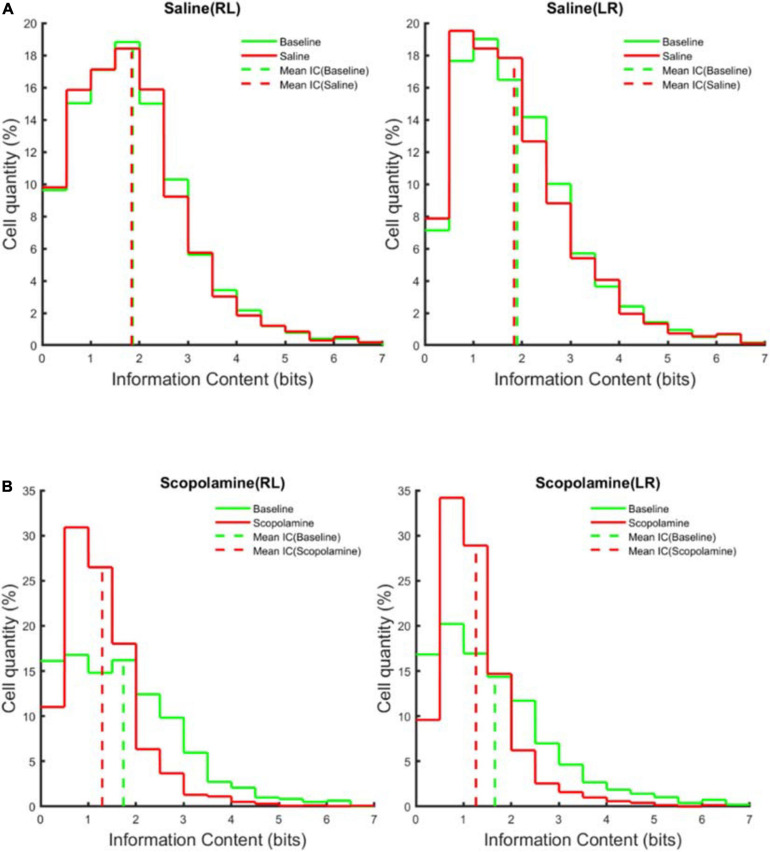
The average information content distribution of the cells measured across five animals before (green solid line) and after (red solid line) **(A)** saline or **(B)** scopolamine (1 mg/kg) injection in right-to-left (RL) and left-to-right (LR) running directions. The dashed vertical line represents the mean information content.

Head direction plays an important role in rodent spatial navigation (McNaughton et al., 1983), so we analysed the data separately on the running trials of right-to-left (RL) and left-to-right (LR). Prior to the injection of scopolamine, 354 ± 26 place cells were observed in each animal in the RL and 323 ± 24 place cells in the LR running sessions. After the administration of scopolamine, the numbers reduced to 203 ± 32 (RL) and 203 ± 33 (LR). The percentage of place cells in both running directions significantly declined after scopolamine injection (RL: 57.63 ± 8.10% and LR: 62.73 ± 9.06% relative to baseline) compared with saline (RL: 109.43 ± 2.10% and LR: 100.49 ± 3.43% relative to baseline) (two-way repeated-measures ANOVA; main effect of injection, *F*1,4 = 22.19, *P* = 0.009; main effect of running direction, *F*1,4 = 0.26, *P* = 0.637; interaction, *F*1,4 = 5.60, *P* = 0.077; post hoc test saline versus scopolamine: RL, *P* = 0.0099; LR, *P* = 0.0477; Figure 3A). Spatial information content (SIC), a measure of location sensitivity of neurons was then quantified. The average SIC of all cells observed was significantly lower in scopolamine treated mice (RL: 74.35% ± 4.91% and LR: 75.14% ± 5.47% relative to baseline) than saline treated mice (RL: 102.44% ± 4.36% and LR: 99.00% ± 3.18% relative to baseline) (two-way repeated-measures ANOVA; main effect of injection, *F*1,4 = 17.11, *P* = 0.014; main effect of running direction, *F*1,4 = 0.46, *P* = 0.535; interaction, *F*1,4 = 5.68, *P* = 0.076; post hoc test saline versus scopolamine: RL, *P* = 0.0249; LR, *P* = 0.0036; Figure 3B). The absolute SIC values were 1.71 ± 0.17 bits and 1.63 ± 0.13 bits on RL and LR running directions prior to scopolamine treatment, decreasing to 1.29 ± 0.20 bits and 1.25 ± 0.18 bits after injection. Additionally, the number of neurons with high SIC (>2 bits) was significantly reduced in the scopolamine group (RL: 22.37% ± 4.59% and LR: 29.51% ± 7.21% relative to baseline) compared with the control group (RL: 111.56% ± 12.02% and LR: 99.65% ± 6.93% relative to baseline) (two-way repeated-measures ANOVA; main effect of injection, *F*1,4 = 30.89, *P* = 0.005; main effect of running direction, *F*1,4 = 0.28, *P* = 0.623; interaction, *F*1,4 = 23.58, *P* = 0.008; post hoc test saline versus scopolamine: RL, *P* < 0.0087; LR, *P* < 0.0133; Figures 4A,B).

### Neuronal Ensemble Properties Were Impaired by Scopolamine

**FIGURE 5:**
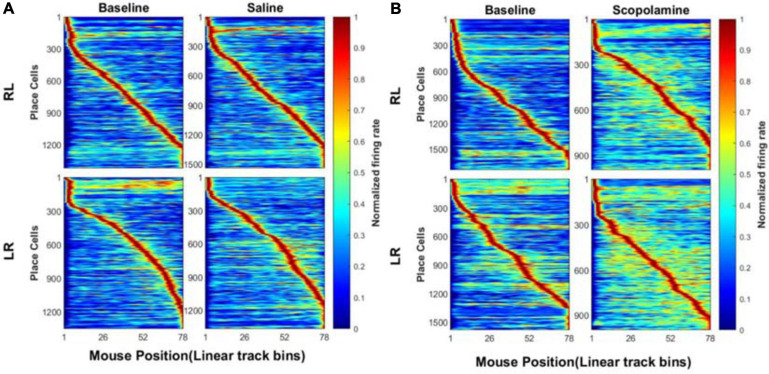
Normalised place field map (see section “Methods”) when the animals run on the linear track before and after the administration of **(A)** saline or **(B)** scopolamine (1 mg/kg) on both running directions. The bin size was 2 cm. The location sensitivity of the place cells in saline group was very stable, while it decreased considerably after scopolamine administration.

**FIGURE 6:**
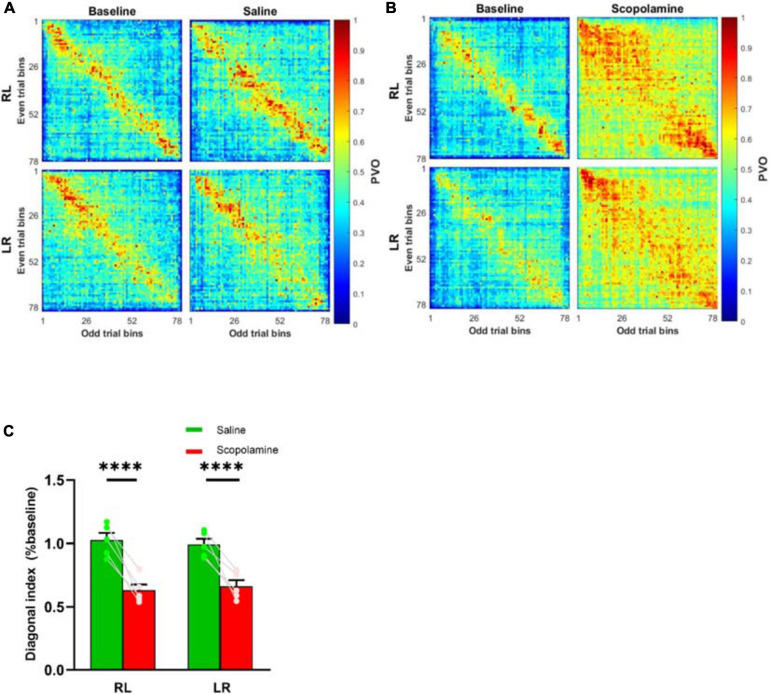
Normalised population vector overlap (PVO) of the place cells between odd trials and even trials before and after the administration of **(A)** saline or **(B)** scopolamine (1 mg/kg) in both running directions. The overlap along the diagonal in baseline indicated spiking at the same bins, and this feature disappeared after scopolamine injection. **(C)** The diagonal index variation measured during RL and LR running trials. ^****^P < 0.0001 represents the significant difference between scopolamine and saline groups.

**FIGURE 7:**
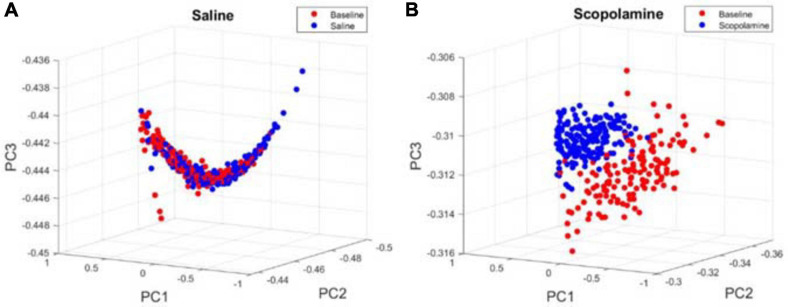
Examples of principal component space for the results of the spike-train epochs after applying unsupervised autoencoder. Each circle represents a 2.5 s data segment of recordings at baseline (red) or **(A)** saline/**(B)** scopolamine (blue).

In addition to the individual neuron properties described above, we explored the effects of scopolamine on neural ensemble activity by calculating the animal’s place field map (see section “Methods,” Figures 5A,B). Normally the firing of place cells displayed very high stability within the place fields. Before injecting scopolamine, place cells displayed relatively consistent firing locations in both running directions. Although most of the place cells still showed a certain degree of place sensitivity after scopolamine injection, it was greatly reduced compared with baseline and the neural off-field firing became stronger. In order to quantify the level of “ensemble stability,” we analysed the population vector overlap (PVO) between the odd trials and even trials data within every session. This revealed the degree of overlap of the ensemble activity in these two different conditions. In Figures 6A,B, a clear diagonalisation feature during the baseline assessment in both running directions is demonstrated, showing a specific and consistent ensemble firing pattern in both even and odd trials. After scopolamine injection, this diagonalisation feature was greatly reduced. The diagonal index was significantly higher in saline treated mice (RL: 102.60% ± 5.61% and LR: 99.07% ± 4.46% relative to baseline) than scopolamine treated mice (RL: 62.88% ± 4.64% and LR: 66.24% ± 4.74% relative to baseline) (Figure 6C; two-way repeated-measures ANOVA; main effect of injection, *F*1,4 = 45.56, *P* = 0.003; main effect of running direction, *F*1,4 = 0.003, *P* = 0.956; interaction, *F*1,4 = 7.57, *P* = 0.051; *post hoc* test saline versus scopolamine: RL, *P* < 0.0001; LR, *P* < 0.0001). The difference of the firing patterns was compared separately by using the autoencoder described above. After data dimensionality reduction and extraction of the most significant information from the input spike-train epochs, epochs in the scopolamine treated groups were clearly separated into two clusters, while the saline control group converged (Figures 7A,B).

### Scopolamine Decreased Decoding Accuracy

**FIGURE 8:**
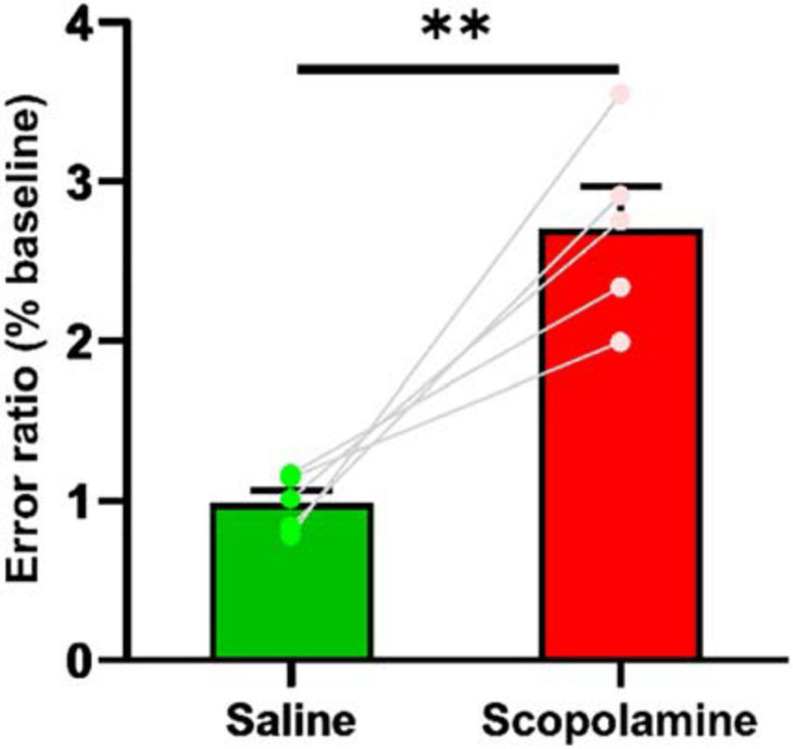
The decoding error ratio. The scopolamine treated mice had higher error ratio. ^∗∗^*P* < 0.01 represents the significant difference between scopolamine and saline groups.

We analysed the animal’s neural decoding accuracy by using a Bayesian decoder to predict the animal’s position (see section “Methods”). Only the activity of place cells was used to train the decoder. The decoding error ratio in scopolamine group (270.74% ± 26.48% relative to baseline) was significantly higher than that in saline group (98.79% ± 7.80% relative to baseline; paired *t*-test, *P* = 0.007). Prior to the injection of scopolamine, the average estimation error was 1.45 ± 0.14 cm/frame, increasing to 4.02 ± 0.61 cm/frame following injection (Figure 8).

### Effect on Running Speed

**FIGURE 9:**
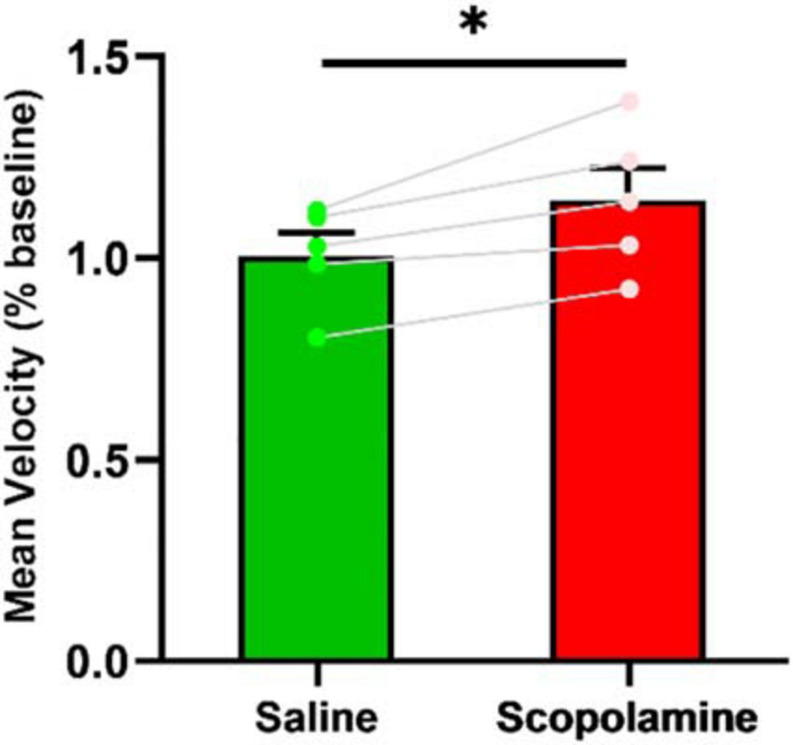
The animal’s running speed. The scopolamine treated mice had higher running velocity. ^∗^*P* < 0.05 represents the significant difference between scopolamine and saline groups.

The animal’s average running speed was measured using all the data on both running directions (except the bins on each end of the linear track). The scopolamine treated mice had a slightly higher running velocity (114.32% ± 8.10% relative to baseline) compared with saline treated ones (100.01% ± 5.66% relative to baseline; paired *t*-test, *P* = 0.02; Figure 9).

### A Pyramidal/Interneuron Conductance-Based Model of CA1 Place Cells Recapitulates Reduced Cell Firing and Place Cell Specificity Through M-Current Modulation

**FIGURE 10:**
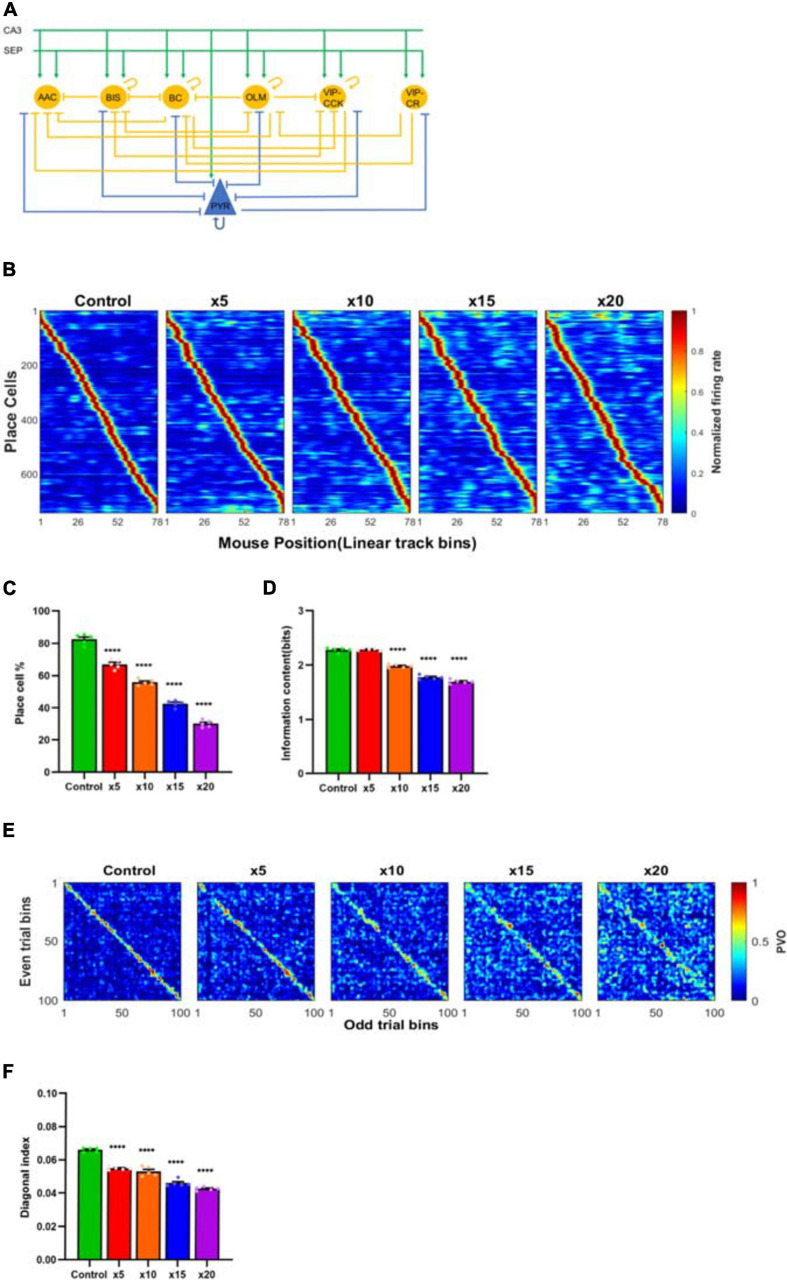
**(A)** Schematic of the hippocampal network model. The model contains connectivity of pyramidal cells (PYR), basket cells (BC), axoaxonic cells (AAC), bistratified cells (BIS), oriens lacunosum-moleculare cells (OLM), vasoactive intestinal peptide-cholecystokinin cells (VIP-CCK) and vasoactive intestinal peptide–calretinin cells (VIP-CR). The afferent inputs come from hippocampal CA3 and medial septum (SEP). **(B)** The decreased neural spatial sensitivity was observed in the place field maps with enhancements of M-channel conductances of pyramidal cells by five (×5), ten (×10), fifteen (×15) and twenty (×20) times separately. The M-channel conductances of SST+ and PV+ interneurons were enhanced to a level reproducing the neural firing reduction observed experimentally. Enhancements of M-channel conductances of pyramidal cells significantly reduced **(C)** place cell quantity and **(D)** neural information content in all groups except ×5 group, which only impacted place cell quantity. **(E,F)** The “diagonalisation feature” of ×5, ×10, ×15 and ×20 groups significantly reduced compared with the control group. ^****^P < 0.0001 represents the significant difference treatment groups and control group.

Modelling scopolamine effects by enhancement of M-channel conductance impaired spatial firing patterns significantly with decreased neural firing rate, quantity of place cells and SIC, leading to less consistent spatial firing patterns (one-way ANOVA, *F*_4,20_ = 3384, *P* < 0.001; Figure 10B). The percentage of place cells in the control group was 82.47% ± 1.43%, while it decreased to 66.92% ± 1.26% in ×5 group (*P* < 0.0001); 55.84% ± 0.83% in x10 group (*P* < 0.0001); 42.61% ± 0.93% in x15 group (*P* < 0.0001) and 30.15% ± 0.96% in x20 group (*P* < 0.0001; Figure 10C). Similarly, the SIC also showed a decreasing trend with respect to the enhanced M-channel conductance (one-way ANOVA, *F*_4,20_ = 337.6, *P* < 0.0001). SIC was 2.28 ± 0.02 bits in both control and ×5 (*P* > 0.99) groups, but it decreased to 1.98 ± 0.01 bits in ×10 group (*P* < 0.0001); 1.77 ± 0.02 bits in ×15 group (*P* < 0.0001) and 1.69 ± 0.02 bits in ×20 group (*P* < 0.0001, Figure 10D). In Figure 10E, a clear diagonalisation feature was observed in the control group, while this feature was greatly reduced in the other groups (one-way ANOVA, *F*_4,20_ = 132.8, *P* < 0.0001). The diagonal index was 0.66×10^−1^±0.54×10^−3^ in the control group, significantly reduced to 0.55×10^−1^±0.43×10^−3^ in x5 group (83.09% ± 1.00% of the control level, *P* < 0.0001), 0.53×10^−1^±0.13×10^−3^ in x10 group (80.24% ± 1.59% of the control level, *P* < 0.0001), 0.46×10^−1^±0.89×10^−3^in x15 group (69.62% ± 1.27% of the control level, *P* < 0.0001), and 0.42×10^−1^±0.49×10^−3^ in x20 group (64.47% ± 0.83% of the control level, *P* < 0.0001; Figure 10F).

## Discussion

We have studied the effect of systemic administration of scopolamine on the activity of hippocampal place cells in mice running in a linear track. The main effects observed were a notable reduction in the activity of neurons throughout the CA1 region, as well as greatly impaired spatial resolution and associated metrics of place cells.

### The Effects of ACh on Memory

Previous studies have hypothesised that Ach facilitates memory encoding through enhancement of the signal-to-noise ratio, improving theta rhythm modulation, increasing persistent spiking or potentiating afferent input (Hasselmo, 2006; Dannenberg et al., 2017). Pharmacological blockade of Ach transmission by mAChR antagonists atropine or scopolamine impairs the acquisition of new memories. In this study, we did not attempt to study encoding events, but rather the effects of mAChR blockade on neural activity as well as ensembles that have previously encoded spatial information in CA1, using miniscope neuronal calcium signals. Scopolamine had quite striking effects on cellular and ensemble behaviour and disrupted place cell specificity. The animals had been trained to traverse the linear track for multiple times before the experiment and given 30 min to become familiar with the environment again before the recording. The spatial memory should have already been fully encoded by previous training as the results of the neural firing rate, spatial information content, place cell quantity, decoding accuracy and the PVO in the saline control group are quite stable before and after the injection. We did not formally behaviourally test recall but given the disruption to the encoded spatial information demonstrated, we expect recall would be likely to be impaired to some extent. Whilst the animals were still capable of recalling the spatial position of the food reward, the retention of this basic ability does not exclude the possibility of some degradation of recall. This could be validated with the use of more accurate methods such as the Morris or Barnes maze. Huang et al. (2011) documented impairment of memory recall in scopolamine treated mice in the Morris maze, consistent with this hypothesis.

### The Effects of mAChR Blockade on Neural Cellular Activity and Network Dynamics

The reduction in both the quantity of detected cells, average neuron firing rate and cell properties implied impairment of neuronal activity at the network level likely related to the pharmacological effects of muscarinic blockade. One mechanism by which the reduction in cellular activity could be explained is by the effect of muscarinic receptors on potassium channels, especially the M-current. The M-current is carried by Kv7 subtype channels inhibited by muscarinic receptors that are expressed at high density at the axon hillock - a specialised area of neurons modulating cell firing (Shah et al., 2008). ACh normally inhibits this current and increases neuronal firing. Hence, scopolamine would be expected to reduce overall neural activity. Previous studies have identified the role of ACh in regulating neural excitability by modulating the M-current (Benardo and Prince, 1982; Cole and Nicoll, 1983). There are other mechanisms by which muscarinic receptors could decrease neuronal activity by enhancing potassium conductance, such as the calcium-activated non-selective cationic current, which is likely to modulate neural persistent firing but less strongly than M-current (Knauer et al., 2013). The place field maps became much less precise after the administration of scopolamine, with increased neuronal activity outside the place field. This is in consistent with the results of Brazhnik et al. (2003, 2004) using electrophysiologically identified CA1 place cells in rats. Newman et al. (2017) and Venditto et al. (2019) found that scopolamine significantly reduced phase precession of spiking relative to the local field theta and the spatial information of the cells, but the spatial tuning of the cells that had preserved place fields remained stable. In the current experiments, we found that there was greatly increased variability of neuronal activity in the observed neurons, both in place fields and surrounding areas which was quantified by calculating the PVO between odd and even trials. This variability was manifested as greatly reduced “diagonalisation” after blocking mAChRs, indicating weakened temporal stability. Additionally, these changes were further explored with the autoencoder algorithm. We found that the encoder effectively separated the spike-train epochs before and after mAChRs blockade, implying the effects of scopolamine on the neural network may be reflected in a higher dimensional metric space. To further explore the impairment of ensemble spatial encoding, we used a naive Bayesian classifier to estimate the animal’s position in the linear track. After the administration of scopolamine, the position estimation deteriorated with a great increase in error rate, consistent with the PVO changes.

### The Effects of ACh on Animal Behaviour

We used time of onset and drug doses similar to previous studies (Klinkenberg and Blokland, 2010). We found the average running speed increased slightly after injecting scopolamine, differing from previous findings (Douchamps et al., 2013; Newman et al., 2017). This may be due to a higher range dose used, complex pharmacological effects of muscarinic blockade or possibly a difference between mice and rats. An increase in locomotion speed was observed in Bushnell (1987) and Thomsen (2014) with dose-dependent enhancement in mobility, with about 25 and 50% increase with 1 mg/kg respectively. Additionally, M1 mAChR knock-out mice were found to have increased locomotor activity (Miyakawa et al., 2001).

#### Hippocampal CA1 Network Model

Place cells were simulated based on previously described hippocampal network models (Turi et al., 2019; Shuman et al., 2020). In these models, the synaptic input to the network originates from entorhinal cortex (EC), CA3 and medial septum. As low ACh level is believed to shift network dynamics toward memory retrieval and suppress the memory encoding process (Dragoi et al., 1999; Hasselmo, 1999), we deleted the EC-CA1 connectivity in our model to simulate the retrieval of spatial information. The blockade of mAChR normally enhances the M-current at the cellular level, but exceptions have also been reported (Carver and Shapiro, 2019). In the simulation study, we hypothesised that a major effect of scopolamine would be the enhancement of the M-current. Inhibition of the M-current has been identified to modulate neuronal firing rates in pyramidal cells (Shah et al., 2008; Honigsperger et al., 2015). In hippocampal interneurons, ACh affects firing properties (Dannenberg et al., 2016). Both PV+ and SST+ interneurons produce a range of responses to muscarinic agonists (Xiang et al., 1998; McQuiston and Madison, 1999). Immunohistochemistry results show the expression of M-channel on PV+ interneurons (Cooper et al., 2001; Lawrence et al., 2006), and mAChRs are observed on PV+ interneurons by Cea-del Rio et al. (2010) and Yi et al. (2014). The model reproduced the reduced place cell number and information content, sparser place field map and impaired stability of spatial representation revealed by hippocampal neural ensemble (PVO map). Differences occurred between the model-derived place field map and the one that empirically observed, possibly due to the simplifications inherent in the model.

#### Miniscope Methodology

Electrophysiological studies commonly use single electrode or electrode arrays to study the activities of brain networks with the help of associated spike sorting algorithms. These algorithms are usually based on the properties of the neural firing rate, spike duration, and the autocorrelation function (Csicsvari et al., 1999; Rossant et al., 2016) in order to distinguish the spikes generated by different neurons. Hence, spatial resolution is not always guaranteed. Another issue with this technique is the limited quantity of cells detected and potential variability over time. In contrast, the miniscope provides a larger area of view, generally displaying the stable activity of hundreds of CA1 neurons that is particularly suitable for the study of network activity. Nonetheless the slow dynamics of the calcium activity, the temporal resolution of calcium imaging cannot match that of electrophysiological recording. Pharmacological manipulation by muscarinic antagonists such as scopolamine might possibly directly affect the calcium conductance of the neurons. A relevant question is whether the effects seen with calcium imaging may just result from the impact of muscarinic antagonism on calcium transients, rather than neuronal activity as such. Interestingly, muscarinic agonists have been found to inhibit calcium currents in hippocampal pyramidal cells (Gahwiler and Brown, 1987), and the calcium signal in response to a single action potential is reduced during muscarinic stimulation (Power and Sah, 2002), implying muscarinic blockade could potentially enhance calcium signal in some circumstances. Nonetheless, several studies have shown that muscarinic agonists enhance intracellular calcium transients, especially in dendrites and with multiple action potentials (Tsubokawa and Ross, 1997; Power and Sah, 2002), potentially causing the apparent reduction in neuronal activity we report. While it is impossible to absolutely exclude such direct effects, we think it is likely the cellular activity recorded by the miniscope does substantially reflect true neuronal and network activity affected by scopolamine, as similar effects were seen with electrophysiological recordings by Brazhnik et al. (2003, 2004). The virus injected into hippocampal CA1 is non-specifically expressed in both pyramidal cells and interneurons; both play essential roles in the animal’s spatial navigation via different mechanisms of action (Gloveli, 2010). It would be of great interest to observe interneuron dynamics separately in response to scopolamine. However, the current miniscope recording system cannot technically distinguish them. A possible way to overcome this issue is to inject another specific-targeting virus to mark the interneurons with a different colour of fluorophore and replace the stimulation mono-colour light source to multi-colour (Aharoni et al., 2019). Both enhancement of the recording system and experimental design deserve further study.

In summary, our results demonstrate that mAChRs have an important role in regulating spatial memory and scopolamine has a strong disruptive effect on the decoding of neural correlates of spatial memory, most likely related to the blockade of mAChR. This is plausibly modulated through increasing M-current amplitude with reduction of neuronal excitability and impairment of network function. Taken as a whole, our results are in accordance with the Hasselmo’s proposal (2006) of a primary effect of Ach being to enhance the signal-to-noise ratio in neural networks. It will be particularly interesting in future studies to directly observe interneuron dynamics, as well as directly assess spatial information decoding.

## Data Availability Statement

The raw data supporting the conclusions of this article will be made available by the authors, without undue reservation.

## Ethics Statement

The animal study was reviewed and approved by Florey Animal Ethics Committee.

## Author Contributions

DS: project conception, drafting and revision of work, data acquisition, calcium imaging, and modelling experiments analysis. RU: experimental design and critical manuscript revision. CF: project conception, data analysis, and critical manuscript revision. All authors have read and approved the final manuscript.

## Conflict of Interest

The authors declare that the research was conducted in the absence of any commercial or financial relationships that could be construed as a potential conflict of interest.
